# EXPRESSION OF CONCERN: Beneficial Effects of Co‐Ultramicronized Palmitoylethanolamide/Luteolin in a Mouse Model of Autism and in a Case Report of Autism

**DOI:** 10.1111/cns.70169

**Published:** 2024-12-09

**EXPRESSION OF CONCERN:** B. Bertolino, R. Crupi, D. Impellizzeri, G. Bruschetta, M. Cordaro, R. Siracusa, E. Esposito, and S. Cuzzocrea, “Beneficial Effects of Co‐Ultramicronized Palmitoylethanolamide/Luteolin in a Mouse Model of Autism and in a Case Report of Autism,” *CNS Neuroscience & Therapeutics* 23, no. 1 (2017): 87–98, https://doi.org/10.1111/cns.12648.

This Expression of Concern is for the above article, published online on 04 October 2016, in Wiley Online Library (http://onlinelibrary.wiley.com/), and has been issued by agreement between the journal Editor in Chief, Xiaoming Hu; and John Wiley & Sons Ltd. The publisher received a report from a third party which indicated that a set of beta‐actin control bands in a western blot was duplicated between Figures 2B and 2H. Further investigation by the publisher revealed duplications of the beta‐actin bands within and between Figures 2 and 4. The authors responded to the publisher's inquiry and request for original data and explained that the same western blot membranes were stripped and re‐probed with different antibodies in order to minimize the number of gels and save sample materials, which explains the duplicated beta‐actin bands. However, the authors did not supply all of the genuine original data for the western blot. The Expression of Concern has been agreed on because the editor and publisher are not able to confirm the validity of the results in Figure 2. The authors did not respond to our notice regarding the Expression of Concern.

